# WEARABLES IN LONG-TERM DEMENTIA RESEARCH: A MIXED METHOD STUDY OF USER EXPERIENCES AND SUPPORT NEEDS

**DOI:** 10.1093/geroni/igae098.3187

**Published:** 2024-12-31

**Authors:** Colleen Peterson

**Affiliations:** University of Michigan Transportation Research Institute, Ann Arbor, Michigan, United States

## Abstract

Passive wearables data collection may be specifically beneficial to aging research featuring dementia populations, who have caregiving and cognitive burdens that can make study participation and reliable data collection more difficult, especially as dementia progresses. This three-phase project aims to inform best practice recommendations to enhance recruitment and adherence in long-term wearables research featuring this population. Based on our systematic review and preliminary in-house data testing, we selected three wearables offering different capabilities and form (from Garmin, Pulse HR, and AngelSense) to test real world usability, data quality, and support needs. This is the first study to recruit persons living with dementia and their caregivers to evaluate multiple devices outside of a laboratory or focus group setting (N=12 dyads). The person living with dementia assented to wearing each wearable for two weeks. Their caregiver rated many facets of each device following its use with the Quebec User Evaluation of Satisfaction with Assistive Technology measure. Open-ended questions and a cumulative semi-structured interview provided context and in-depth comparative perspectives of their experiences in the study. Wearable durability, simplicity, and data availability/monitoring capacity were important to participant favorability and adherence. Technical help and check-ins were also deemed highly valuable. Data indicate how the devices suited the dyads’ needs or caused issues, as well as how study staff could better support ongoing use. Collectively, our findings suggest ideal criteria to guide wearable selection and key protocol factors that can enhance participant recruitment and adherence in long-term dementia research.

